# Correlating Patient Symptoms and CT Morphology in AI-Detected Incidental Pulmonary Embolisms

**DOI:** 10.3390/diagnostics15131639

**Published:** 2025-06-27

**Authors:** Selim Abed, Lucas Brandstetter, Klaus Hergan

**Affiliations:** General Hospital Salzburg, Müllnerhauptstrasse 48, 5020 Salzburg, Austria; brandy_lucas@gmx.at

**Keywords:** incidental pulmonary embolism, artificial intelligence, natural language processing, clinical correlation

## Abstract

**Background/Objectives**: Incidental pulmonary embolisms (IPEs) may be asymptomatic and radiologists may discover them for unrelated reasons, and they can thereby go underdiagnosed and undertreated. Artificial intelligence (AI) has emerged as a possible aid to radiologists in identifying IPEs. This study aimed to assess the clinical and radiological significance of IPEs that a deep learning AI algorithm detected and correlate them with thrombotic burden, CT morphologic signs of right heart strain, and clinical symptoms. **Methods**: We retrospectively evaluated 13,603 contrast-enhanced thoracic and abdominal CT scans performed over one year at a tertiary care hospital using a CE- and FDA-cleared AI algorithm. Natural language processing (NLP) tools were used to determine whether IPEs were reported by radiologists. We scored confirmed IPEs using the Mastora, Qanadli, Ghanima, and Kirchner scores, and morphologic indicators of right heart strain and clinical parameters such as symptomatology, administered anticoagulation, and 6-month outcomes were analyzed. **Results**: AI identified 41 IPE cases, of which 61% occurred in oncologic patients. Most emboli were segmental, with no signs of right heart strain. Only 10% of patients were symptomatic. Thrombotic burden scores were similar between oncologic and non-oncologic groups. Four deaths occurred—all in oncologic patients. One untreated case experienced the recurrence of pulmonary embolism. Despite frequent detection, many IPEs were clinically silent. **Conclusions**: AI can effectively detect IPEs that are missed on initial review. However, most AI-detected IPEs are clinically silent. Integrating AI findings with morphologic and clinical criteria is crucial to avoid overtreatment and to guide appropriate management.

## 1. Introduction

An incidental pulmonary embolism (IPE) is defined as a filling defect in one or more pulmonary arteries in a CT scan performed for reasons unrelated to suspected pulmonary embolism (PE) [1]. Although often clinically silent, IPEs are not necessarily benign. IPEs often go undetected and can be found in asymptomatic patients on autopsy [2,3]. The frequency of IPE is estimated to be between 2.6 and 3.4% [4,5]. Some researchers have suggested an increase in the frequency of IPEs, probably due to an increasing number of CT scans performed in hospitals [6]. IPEs are more common in certain patient populations such as oncological patients and are for the most part unsuspected [7]. In one study, Sørensen et al. found that one-year survival was 32% in cancer patients with venous thromboembolism compared to 62% survival in those without venous thromboembolism [8]. Untreated IPE often leads to recurrence and high mortality if left untreated, leading to a poorer prognosis in patients with cancer [4].

Despite these risks, IPEs are often subtle and easily overlooked during routine review by radiologists, especially when they involve peripheral pulmonary arteries. How can we improve the detection rate to enable adequate treatment in patients? Could AI support radiologists in the detection of IPE? And if so, how do we interpret the algorithm’s findings in a clinical context and what are the clinical implications?

AI may aid as an adjunctive tool and support radiologists in the detection of IPE [9]. Deep learning algorithms have demonstrated the ability to identify targets that may be missed on initial radiological interpretation. Batra et al. demonstrated an AI algorithm with a highly negative predictive value and a moderately positive predictive value, detecting some IPEs missed by radiologists [10]. Therefore, using an AI algorithm as a second-look tool may prove useful. Bach et al. examined imaging characteristics and embolus burden in unreported PE in oncologic patients. They found that nearly one third of all PE events were unsuspected, of which most went unreported [7]. Another factor that leads to IPEs being overlooked is when patients are asymptomatic [5,11]

However, while the diagnostic performance of AI for PE detection is well established, its clinical impact remains unclear. Research on the clinical and morphological significance of AI-detected IPEs and their correlation with patient symptoms is sparse. Deniz et al. found that the detection of PE in patients with apparent clinical signs is relatively easy, unlike with patients with subtle or no symptoms. Radiologists often do not detect PEs in asymptomatic patients when contrast-enhanced thoracic CT is performed for disease staging or therapy response monitoring [12]. To our knowledge, this is the first study examining embolic burden and CT morphologic criteria of right heart strain in IPEs detected with AI and correlating them to patient symptoms.

We used a medical-grade deep learning algorithm to analyze all CT scans performed at our department to detect IPEs. Using natural language processing (NLP), we identified cases of IPE that radiologists had not reported. We then assessed thrombotic burden using four scoring systems (Mastora, Qanadli, Ghanima, and Kirchner), analyzed several CT morphologic criteria to determine right heart strain, and reviewed clinical data to correlate imaging findings with patient symptoms and outcomes. The primary objective of this study was to investigate whether AI-detected IPEs that were initially unreported demonstrated CT morphological signs of right heart strain and if the patients’ symptoms matched the embolic burden. We did not formulate a strong a priori hypothesis since this paper is exploratory in nature. Our aim was to better understand the clinical relevance of AI-detected IPEs using objective criteria.

## 2. Materials and Methods

### 2.1. Study Design and Setting

We conducted this retrospective study at a tertiary-care university hospital from December 2022 to December 2023. The primary objective was to evaluate IPEs detected with a deep learning AI algorithm and correlate these findings with clinical and morphological criteria. An IPE is defined as a filling defect in one or more pulmonary arteries on a CT scan performed for reasons unrelated to suspected PE. This excludes all CT pulmonary angiogram (CTPA) examinations.

### 2.2. Dataset

From December 2022 to December 2023, a total of 13,603 thoracic and abdominal CT scans were performed at the university hospital. These scans included contrast-enhanced CTs obtained for various indications unrelated to suspected PE. We excluded all CTPA examinations from the study. Scans were acquired according to standard department protocols, with bolus timed intravenous contrast administration and standard reconstruction parameters. The AI-algorithm operated 24/7 including all out-of-hour shifts, ensuring consistent triage and case processing across the full period. The dataset included a diverse population, with both oncological and non-oncological cases.

### 2.3. Study Design and AI Tool Implementation

This study employed a commercially available AI-based image analysis software (Aidoc Medical, Tel Aviv, Israel) to evaluate conventional contrast-enhanced CT examinations performed at our institution. The AIDOC algorithm is based on a deep convolutional neural network (CNN). The algorithm was trained and validated on a substantial dataset of CT scans over multiple institutions, specifically trained to detect acute IPEs, and designed for prioritization and triage in clinical workflows. The software was previously validated using tens of thousands of CT examinations and optimized for study-level classification. The AI tool was cleared by both the Conformité Européenne (CE) and the U.S. Food and Drug Administration (FDA).

### 2.4. Integration with Clinical Workflow

**Figure 1:**
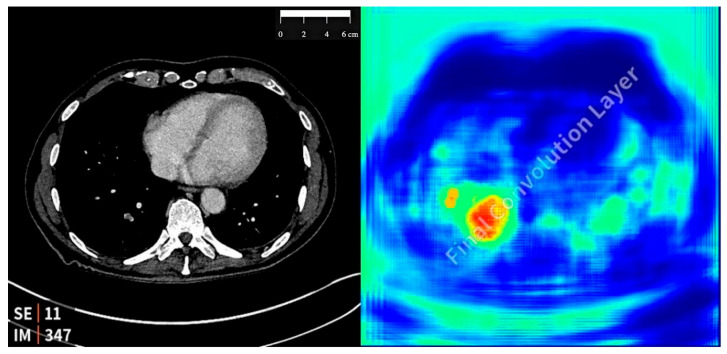
Axial CT of an AI-detected PE on the left with an AI-generated heatmap on the right.

The AI software was integrated with the institution’s Picture Archiving and Communication System (PACS) and Radiology Information System (RIS). It automatically analyzed eligible imaging studies immediately after acquisition. The algorithm classified examinations as positive or negative for IPE. For cases classified as positive, a heatmap was generated, highlighting the regions of the images with suspected emboli. See Figure 1 for an example of an AI-detected PE and the generated heatmap. Positive results were flagged in the radiologist’s worklist in the RIS with a bright color for prioritization.

We measured the pulmonary artery, the aorta, the ratio of the pulmonary artery to the aorta, the superior vena cava, and the azygous vein and analyzed the scans for any degree of septum bowing. We noted which arteries were affected by PEs and whether a partial or complete occlusion was present. Complete occlusion is defined as a 100% filling defect of any order in the pulmonary artery. We then used the Mastora, Qanadli, Ghanima, and Kirchner scores to grade the embolic burden.

The Mastora scoring system offers a detailed evaluation of the total embolic burden by grading embolic obstruction in five mediastinal, six lobar, and twenty segmental arteries. These arteries are then scored for the degree of obstruction due to embolism on a scale from 0 to 5 (0 = 0%; 1 = 1–24%; 2 = 25–49%; 3 = 50–74%; 4 = 75–99%; 5 = 100%). The sum of the scores gives a global and comprehensive obstruction score with the maximum value of 155. This scoring system is useful for global assessment across all pulmonary artery territories and for detecting subtle difference in clot load.

The Qanadli scoring system focuses on segmental artery involvement by assigning 1 point for partial obstruction and 2 points for complete obstruction of each segmental artery (20 total and 10 per lung), with a maximum score of 40. It emphasizes functional obstruction and can be correlated with right ventricular dysfunction.

The Ghanima score is based on the most proximal extension of the PE relative to the main pulmonary arteries and divides the pulmonary arterial tree into four anatomical levels (mediastinal, lobar, segmental, and subsegmental). The score is then calculated by awarding four points for extension into mediastinal arteries, three points for lobar arteries, two points for segmental arteries, and one point for subsegmental arteries in each one of the four levels. The score emphasizes anatomical severity rather than cumulative clot volume.

The Kirchner score is a modified and simplified version of the Miller score, where 1 point is assigned for embolic involvement in each of the 16 segmental arteries, making the maximum score 16. It is a binary measurement only focusing on the presence and absence of an embolus in segmental arteries.

By applying all four scoring systems, we aimed to capture both the extent and anatomical severity of embolic burden, as well as function obstruction.

### 2.5. Clinical Correlation with Patient Symptoms

Several factors to corelate clinically were determined. The level of expertise or profession of the relevant medical professional was determined. We analyzed whether the patient received anticoagulatory therapy, and if so, what kind of therapy. We defined patients as asymptomatic if they had no symptoms relating to IPEs such as chest pain, tachypnea, hypoxia, cough, or hemoptysis. If any of these symptoms were present, then patients were defined as symptomatic. Even mild symptoms such as mild dyspnea and pleuritic pain were included. We checked if symptoms pertaining to PEs were listed in the CT request, patient notes, round notes, or other documents within the patient records, and analyzed if there was a possible confounding factor leading to dyspnea. We went through patient records and the PACS to check if patients had a previous PE, or if patients suffered from a subsequent PE within the next six months and if that PE was the same one previously detected by the AI algorithm. Lastly, we checked if patients presented to the emergency department with dyspnea or if they passed away in the following six months.

## 3. Results

### 3.1. Detection of Incidental Pulmonary Embolism

A total of 13,603 CT scans, not including CTPA, in the period between December 2022 and December 2023 were analyzed using the AI algorithm. A board-certified radiologist specialized in cardiac imaging confirmed 41 cases of IPEs as true positives, which equaled 0.3% of all analyzed CT scans. The average age of patients with an AI-detected IPE was 65.3. In total, 25 were female patients and 16 were male patients.

Of these,

- A total of 25 cases (61%) involved oncological patients (15 females and 10 males);
- A total of 16 cases (39%) involved non-oncological patients (10 females and 6 males).

We evaluated the thrombotic burden using the Mastora, Qanadli, Ghanima, and Kirchner scoring systems.

### 3.2. Symptom and CT Morphological Analysis of Oncologic Patients

The average age of the patients with an AI-detected IPE was 66.2.

The average scores for the 25 oncological patients were as follows:

Average Mastora: 151.8;

Average Qanadli: 2.0;

Average Ghanima: 2.8;

Average Kirchner: 1.2.

In total, 20 patients only had an IPE located in a segmental artery, while 2 patients had an IPE located in lobar arteries. No patients had an IPE located in a mediastinal artery. The average ratio of the right ventricle to the left ventricle and the ratio of the common pulmonary artery to the ascending aorta were 0.8. No patients had septum bowing.

Two patients were symptomatic, one of them with a confounding factor (broken rib).

We had one patient who experienced recurrence after not receiving anticoagulation initially. No patients suffered from a previous PE.

The average age of patients treated with and without anticoagulants was 64.6 and 68, respectively. Four patients passed away within six months; their average age was 69.8.

See Table 1 for more information on oncological patients and their respective results.

### 3.3. Symptomatic and CT Morphological Analysis of Non-Oncologic Patients

The average age of patients with an AI-detected IPE was 64.

The average scores for the 16 non-oncological patients were as follows:

Average Mastora: 152.2;

Average Qanadli: 1.9;

Average Ghanima: 2.5;

Average Kirchner: 1.0.

Fourteen patients only had an IPE located in a segmental artery, while two patients had their IPE located in lobar arteries. No patients had an IPE located in a mediastinal artery. The average ratio of the right ventricle to the left ventricle and the ratio of the common pulmonary artery to the ascending aorta were 0.8. No patients had septum bowing.

Two patients were symptomatic, none of them with a confounding factor. No patient suffered from recurrent PE or from a previous PE.

The average age of patients treated with and without anticoagulants was 63.5 and 64.5, respectively. No patients passed away within six months.

See Table 2 for more information on non-oncological patients and their respective results.

### 3.4. Comparison Between Oncological and Non-Oncological Groups

Oncological patients demonstrated similar thrombotic burden scores compared to non-oncological patients, despite their higher rate of IPE detection. Mortality was observed only in the oncological group. Oncological patients more frequently received anticoagulant therapy compared to non-oncological patients.

We performed Fisher’s exact test to compare the different use of anticoagulation between the oncological and the non-oncological groups. The test yielded a *p*-value of 0.53, indicating no statistically significant difference between both patient groups, although the oncological group was more likely to receive anticoagulation (56%) than the non-oncological group (44%). We also performed Fisher’s exact test to compare the difference in appearance of symptoms and the rate of recurrence between the oncological and the non-oncological group. These tests yielded *p*-values of 0.63 and of 0.26, respectively, indicating that there was no statistically significant difference between both patient groups in the rate of symptoms or recurrent PE.

## 4. Discussion

### 4.1. Principal Findings

We did not aim to validate the AI findings and the algorithm’s performance was not the aim of this study. Several studies have calculated sensitivities between 86.6% and 100% and a specificity between 89% and 95.5% for similar algorithms in detecting IPEs [13,14,15,16,17]. The algorithm detected more IPEs in oncological patients than in non-oncological patients. Interestingly, despite the more frequent detection of IPEs in oncological patients, the thrombotic burden scores (e.g., Mastora, Qanadli) were comparable between oncological and non-oncological groups. This suggests that the overall burden of disease may not differ significantly between these patient groups. The Mastora score seems to be the most accurate in determining thrombotic burden, given that it considers all orders of pulmonary arteries. Moreover, given that symptomatic PEs with higher thrombus load are associated with greater dysfunction in the right ventricle, increased pulmonary artery pressure, and increased related mortality, we used CT morphologic criteria to gauge right heart strain [18]. We cannot draw conclusions regarding the correlation between thrombus size and the likelihood of patient symptoms because of the following: Most of the IPEs detected with the AI algorithm were subsegmental. Only four patients had an IPE in a lobar artery, and none of these four patients were symptomatic. Thus, we must acknowledge one of the main limitations of this study, which is the small number of symptomatic patients [5]. Bach et al. found that unsuspected PEs tend to have a lower thrombus load than those with symptomatic PEs. This would explain why IPEs tend to be clinically silent. The absence of CT morphological signs of right heart strain across all patients supports the notion that these emboli were largely clinically insignificant. Conversely, some authors believe that undetected PEs may be precursors to more clinically obvious emboli and should therefore be treated [19]. This may only pertain to symptomatic PEs, as Barrit et al. reported high recurrence and even fatality in untreated symptomatic PE [20]. This highlights the role of AI in flagging emboli that may otherwise escape detection due to a lack of symptoms.

Only 4 out of 41 patients were symptomatic or had confounding factors. This correlates with the predominantly asymptomatic nature of IPEs in our cohort. Our results also highlight the potential for overdiagnosis and overtreatment. While 63% of oncological and 56% of non-oncological patients received anticoagulant therapy, we cannot determine whether all cases warranted treatment. For instance, none of the treated patients in our cohort exhibited signs of right heart strain, and most emboli were small and confined to segmental arteries. These findings emphasize the importance of correlating AI-detected IPEs with clinical and morphological criteria to guide treatment decisions. These findings might also suggest that IPEs detected incidentally with AI, particularly in the absence of symptoms or morphological signs of strain, may not require immediate intervention. Given this information, we should take a closer look at current guidelines, which generally recommend treating all IPEs, whether symptomatic or asymptomatic. Further studies are needed to determine if a subset of truly asymptomatic, low-burden IPEs might safely forgo anticoagulation.

### 4.2. Thrombotic Burden

Ritchie et al. reported that most IPEs missed by radiologists on initial review were peripheral [10]. The IPEs of 4 out of our 41 patients were in a lobar artery, compared to 37 out of 41 patients with an IPE in a segmental artery. This peripheral distribution may partly explain why most IPEs are asymptomatic and frequently missed. This is in accordance with several other studies reporting that IPEs are more likely to be located at the segmental and subsegmental level without an occlusion of the vessel [6,7,21]. Segmental and subsegmental locations are often associated with subtle imaging features that can be overlooked during the initial review. Our findings align with prior studies, demonstrating that most IPEs occur in segmental arteries, locations often associated with subtle imaging features that can be easily overlooked by radiologists during initial review. Gladish et al. reported that patients with PEs in small-sized arteries, which were not detected, were a predisposing factor for deep vein thrombosis or recurrent embolism [22]. Our results support prior findings in that segmental and subsegmental PEs pose a diagnostic challenge, particularly when clinical suspicion is low [23,24]. The AI algorithm successfully identified these emboli, confirming its role as a second-reader tool [25]. As demonstrated in other studies, we also found that all IPEs detected with the algorithm were indeed positive for PE [11]. Given that 37 out of 41 patients had no listed symptoms in their CT referral, this might have been a contributing factor to the IPE being missed by the reporting radiologist initially. Similarly, Krupinski et al. found that unsuspected PEs are more likely to be missed. One must take into account that on average the total embolic burden in IPEs is lower than that in symptomatic PEs [7,21]. Given that most missed IPEs are in segmental and subsegmental arteries, AI may be a helpful adjunct tool with which to avoid missing IPEs on initial review [11,26,27]. This underlines the role of AI in elevating diagnostic sensitivity in routine CT workflows. As imaging volumes continue to rise [28]., such AI support systems may become essential not only for improving detection but also for optimizing patient outcomes through earlier intervention.

### 4.3. Clinical Implications

Previous studies have demonstrated that patients with cancer have a substantially increased risk of dying following an acute thrombotic event compared to patients without cancer [29]. In total, 4 out of 25 oncological patients with an IPE passed away within six months of detection. We find it difficult to attribute causality given the frequent co-occurrence of advanced malignancy and IPEs. One must take into consideration that Sahut et al. found oncological patients with an IPE to have no associated higher risk of mortality compared with control patients without an IPE [30]. This raises the question of whether an IPE in cancer patients serves more as a marker of disease burden than a direct contributor to mortality. Observational studies have also reported no excess morbidity with untreated and unreported IPEs [31]. In particular, subsegmental PEs do not affect survival [1]. This observation aligns with the ongoing debate about whether small, asymptomatic PEs in cancer patients justify full anticoagulation. We find it difficult to separate the burden of the oncological disease from the possible burden of disease from IPE, even though most IPEs do not manifest clinically. All four oncological patients that passed away had clinically occult IPEs presenting no symptoms at detection. To our knowledge, this study is one of the first to systematically correlate AI-detected IPEs with both CT morphologic criteria and patient symptoms, thereby bridging the gap between automated detection and clinical interpretation. By exploring how AI-identified IPEs relate to symptom burden and imaging features such as clot burden and right heart strain, our findings contribute novel insight into the potential role of AI not only as a diagnostic aid but also as a tool for risk stratification.

### 4.4. Implications for Treatment

The American College of Chest Physicians recommends the same initial long-term therapy for both asymptomatic and symptomatic PEs [32]. Conversely, Engelke et al. showed that patients with a clinically unsuspected PE or IPE have a favorable short-term outcome without anticoagulation therapy [33,34,35]. The disparity between guidelines and observational outcomes highlights the ongoing uncertainty surrounding the optimal management of IPEs. We did not report on long-term outcomes or include enough symptomatic patients in our cohort to report on differences between symptomatic and asymptomatic patients with PE. Sorto et al. found that PE progression in one of four patients remained untreated for their IPE [36]. We only found one oncological patient who had recurrent PE on follow-up CT; this patient was not treated initially. The low rate of recurrence in our cohort raises important questions about the necessity and duration of anticoagulation in clinically silent cases. Therefore, we must pose the question of whether we are treating the image or the patient. Clinicians must match AI-driven detection with careful clinical judgment to avoid unnecessary exposure to the risks of anticoagulation. Ultimately, treatment decisions for IPE should consider both imaging characteristics and individual patient context, rather than relying solely on detection. Further research in this field is needed to prevent the overtreatment of IPE, which clinicians might overlook without the help of AI.

### 4.5. Strengths

This study explored the detection of IPEs at the lung bases in contrast-enhanced abdominal CT examinations. One of the key strengths of this study was the use of four distinct scoring systems (Mastora, Qanadli, Ghanima, and Kirchner) to evaluate embolic burden in oncological and non-oncological patients. By employing multiple validated systems, we ensured a comprehensive and robust assessment of embolic burden, minimizing potential biases or limitations associated with any single scoring method. Our study also analyzed CT morphological criteria of right heart strain, including the right-to-left ventricular diameter ratio, interventricular septal bowing, and the reflux of contrast into the inferior vena cava. This comprehensive assessment allowed us to correlate embolic burden with potential hemodynamic consequences. We believe that this is the first study to integrate and correlate embolic burden, CT morphological criteria of right heart strain, and patient-reported symptoms in the context of IPEs detected with AI. By combining these three critical factors, we provide a comprehensive analysis that captures the anatomical, hemodynamic, and clinical dimensions of AI-detected IPEs.

### 4.6. Limitations

We conducted this study at a single tertiary care center, which may limit the generalizability of its findings. Only one radiologist formally verified the AI-detected PE. However, in all cases, the original reporting radiologist was informed of the missed embolism and subsequently confirmed and acknowledged the finding. While this does not replace a blinded second reading, it does represent a form of secondary verification. This study may have suffered from potential selection biases, particularly in the inclusion of oncological patients, who may be more likely to undergo frequent imaging. We did not follow-up on long-term outcomes beyond six months, limiting the conclusions we can draw regarding the progression of untreated PE. Due to the retrospective nature of this study, the rationale for initiating or withholding anticoagulation therapy was not consistently documented in the medical records. As a result, we are unable to determine a standardized decision-making pathway, and treatment decisions appeared to vary based on individual clinical judgment. Out of 13,603 CT scans, only 41 cases of IPE were detected, which represents a very small proportion of the total dataset. Only a few of the patients showed symptoms, making it difficult to draw definitive conclusions about the relationship between symptoms and IPE detected with AI. We could not perform a sufficient statistical analysis of the differences between both groups when correlating patient symptoms and the embolic burden based on their different scores due to the small number of symptomatic patients. Our sample size is too small to detect statistically significant differences in clinical outcomes between oncologic and non-oncologic groups. We cannot exclude the possibility of a type II error, particularly when trends were observed but did not reach statistical significance such as in the use of anticoagulation. Future studies should consider prospective designs, incorporate follow-up outcome data, and explore the integration of AI-detected IPEs into real-time clinical workflows to better assess their impact on patient management.

### 4.7. Future Directions

More research is needed to assess the impact of AI-assisted IPE detection on clinical decision-making and patient outcomes. Incorporating long-term follow-up data would further clarify the natural history of untreated IPEs and inform guidelines for their management.

## 5. Conclusions

Artificial intelligence is a helpful adjunct tool that can be used to detect IPEs missed on initial review. As this was an exploratory study, our aim was to investigate whether objective CT morphologic criteria and symptomatology could help contextualize AI-detected IPEs and guide their clinical relevance.

Correlating patients’ symptoms and CT morphologic criteria with IPEs detected with AI may be a practical solution to avoid overtreatment. **Our findings suggest that not all AI-detected IPEs may require immediate treatment**, especially in the absence of symptoms or morphologic signs of strain. This highlights the potential for **AI-supported triage systems that incorporate both imaging and clinical context to guide treatment decisions more judiciously**.

However, the clinical significance of asymptomatic IPEs, particularly in oncological patients, remains a subject for further investigation. The careful integration of AI findings into clinical workflows will be essential to balance the benefits of improved detection with the risks of unnecessary intervention.

## Figures and Tables

**Table 1:** Oncological patients and their analysis: the arteries and their location involved in the IPE, the four different scores (Mastora, Qanadli, Ghanima, and Kirchner), the ratio of the right ventricle to the left ventricle in the axial plane, the ratio between the pulmonary artery and aorta, the size of the superior vena cava and the azygos vein, if patients received anticoagulation, if the symptoms related to the IPE were listed in the patient records, and if patients suffered from recurrent PE.

Order and Number of Arteries Involved	Location	Mastora	Qanadli	Ghanima	Kirchner	RV/LV	PA/AO	SVC	Azygos Vein	Anti Coagulation	Symptom Listed	Recurrent PE
1 segmental	RL	153	1	2	1	0.77	0.88	12 mm	8 mm	Yes	No	No
1 segmental	RUL	151	2	2	2	0.68	0.77	14 mm	7 mm	Yes	No	No
1 segmental	Lingula	151	1	2	1	0.91	0.63	11 mm	6 mm	Yes	No	No
1 segmental	ML	152	1	2	1	0.86	0.55	11 mm	6 mm	Yes	No	No
1 lobar and 1 segmental	RL	150	8	5	1	0.53	0.89	15 mm	6 mm	No	No	Yes (progress)
1 lobar and 1 segmental	LLL	145	9	10	2	0.69	0.82	14 mm	4 mm	No	No	No
1 segmental	RLL	152	1	2	1	0.78	0.69	12 mm	6 mm	Yes	No	Yes
1 segmental	RLL	153	1	2	1	0.57	0.87	11 mm	7 mm	Yes	Yes (Dyspnea)	No
1 segmental	RLL	153	1	2	1	0.72	0.64	9 mm	6 mm	No	No	Yes
3 segmental	ML	151	2	5	2	0.66	0.81	18 mm	7 mm	Yes	No	No
2 segmental	LLL	149	3	4	3	0.75	0.70	9 mm	7 mm	No	No	No
2 segmental	LLL	149	2	4	2	0.52	0.90	12 mm	6 mm	Yes	No	No
1 segmental	ML	153	1	2	1	0.80	0.72	13 mm	6 mm	Yes	No	No
1 segmental	RL	152	1	2	1	0.74	0.83	13 mm	6 mm	Yes	No	No
1 segmental	RL	153	1	2	1	0.93	0.75	13 mm	5 mm	No	No	No
1 segmental	RL	152	1	2	1	0.59	0.70	17 mm	8 mm	Yes	No	No
1 segmental	RL	153	1	2	1	0.73	0.88	13 mm	7 mm	No	No	No
1 segmental	RL	153	1	2	1	0.88	0.76	12 mm	8 mm	No	No	No
1 segmental	ML	153	1	2	1	0.80	0.72	13 mm	6 mm	No	No	No
1 segmental	RL	153	7	3	0	0.52	0.89	15 mm	7 mm	No	Yes (Dyspnea)	No
1 segmental	RL	153	1	2	1	0.78	0.88	11 mm	7 mm	Yes	No	No
1 segmental	RUL	153	1	2	1	0.56	0.75	9 mm	7 mm	Yes	No	No
1 segmental	RL	152	1	2	1	0.85	0.75	10 mm	6 mm	No	No	No
1 segmental	RL	153	1	2	1	0.56	0.87	9 mm	6 mm	No	No	No
1 segmental	RUL	153	1	2	1	0.82	0.62	12 mm	8 mm	Yes	No	No

**Table 2:** Non-oncological patients and their analysis: the arteries and their location involved in the IPE, the four different scores (Mastora, Qanadli, Ghanima, and Kirchner), the ratio of the right ventricle to the left ventricle in the axial plane, the ratio between the pulmonary artery and aorta, the size of the superior vena cava and the azygos vein, if patients received anticoagulation, if the symptoms related to the IPE were listed in the patient records, and if patients suffered from recurrent PE.

Order and Number of Arteries Involved	Location	Mastora	Qanadli	Ghanima	Kirchner	RV/LV	PA/AO	SVC	Azygos Vein	Anticoagulation	Symptom Listed	Recurrent PE
1 segmental	ML	153	1	2	1	0.78	0.88	13 mm	9 mm	No	No	No
1 segmental	RLL	153	1	2	1	0.63	0.79	8 mm	7 mm	Yes	No	No
2 segmental	RLL	150	2	2	2	0.90	0.97	17 mm	7 mm	Yes	No	No
1 lobar and 2 segmental	RLL	151	2	7	2	0.78	0.65	20 mm	6 mm	No	No	No
1 segmental	Lingula	152	1	2	1	0.83	0.67	12 mm	4 mm	Yes	Yes (Hypoxia/tachycardia)	No
1 segmental	RLL	153	1	2	1	0.81	0.72	15 mm	5 mm	No	No	No
1 segmental	RLL	151	2	1	1	0.41	0.89	14 mm	6 mm	Yes	No	No
1 segmental	RLL	153	1	2	1	0.91	0.71	12 mm	5 mm	No	No	No
1 segmental	RLL	154	1	4	1	0.82	0.71	10 mm	8 mm	No	Yes (tightness in chest)	No
1 segmental	RLL	151	1	2	1	1.1	1.1	25 mm	6 mm	No	No	No
1 segmental	ML	153	1	2	1	0.72	0.62	12 mm	7 mm	No	No	No
1 segmental	LUL	153	1	2	1	0.55	0.87	10 mm	6 mm	Yes	No	No
1 segmental	RUL	153	1	2	1	0.78	0.87	11 mm	5 mm	No	No	No
1 lobar and 1 segmental	RLL	151	7	3	1	0.82	0.78	12 mm	7 mm	Yes	No	No
2 segmental	ML and RLL	151	7	3	0	0.87	0.84	13 mm	9 mm	Yes	No	No
1 Segmental	RUL	153	1	2	1	0.71	0.73	11 mm	7 mm	No	No	No

## Data Availability

The anonymized datasets generated and analyzed during the current study are available from the corresponding author upon request. No formal analysis scripts were used and all statistical analyses were performed manually using standard software tools.

## Abbreviations

The following abbreviations are used in this manuscript:

MDPI	Multidisciplinary Digital Publishing Institute;
DOAJ	Directory of Open Access Journals;
TLA	Three-letter acronym;
LD	Linear dichroism;
IPE	Incidental pulmonary embolism;
PE	Pulmonary embolism;
AI	Artificial intelligence;
NLP	Natural language processing.
